# Isolation and characterization of five novel disulfide-poor conopeptides from *Conus marmoreus* venom

**DOI:** 10.1590/1678-9199-JVATITD-2021-0116

**Published:** 2022-05-18

**Authors:** Ying Fu, Yu Zhang, Shuang Ju, Bokai Ma, Wenwen Huang, Sulan Luo

**Affiliations:** 1 Key Laboratory of Tropical Biological Resources, Ministry of Education, Hainan University, Haikou, China; 2 Key Laboratory for Marine Drugs of Haikou, School of Pharmaceutical Sciences, Hainan University, Haikou, China; 3 Beijing Key Laboratory of Organic Materials Testing Technology & Quality Evaluation, Institute of Analysis and Testing, Beijing Academy of Science and Technology, Beijing, China.; 4 Medical School, Guangxi University, Nanning, China.

**Keywords:** Conopeptides, Disulfide-poor conopeptides, Conus marmoreus, nAChR, Cone snail, *Conus* venom

## Abstract

**Background:**

Conopeptides from cone snail venom have aroused great interest related to the discovery of novel bioactive candidates, due to their excellent prospects for the treatment of various health problems such as pain, addiction, psychosis and epilepsy. In order to explore novel biopeptides, we investigated the structure and function of five novel conopeptides isolated from the venom of *Conus marmoreus* from South China Sea.

**Methods:**

*C. marmoreus* crude venom was prepared, fractionated and purified by HPLC system. The primary sequences of the five novel disulfide-poor conopeptides Mr-1 to Mr-5 were identified by comprehensive analysis of *de novo* MALDI-TOF tandem mass spectrometry and Edman degradation data. In order to investigate their function, these five conopeptides were synthesized by Fmoc-SPPS chemistry, and their biological effects at several heterologous rat nicotinic acetylcholine receptor (nAChR) subtypes (α1β1δε, α3β2, α3β4, α4β2) were determined by electrophysiological technique.

**Results:**

Five novel disulfide-poor conopeptides were identified and named as follows: Mr-1 (DWEYHAHPKPNSFWT), Mr-2 (YPTRAYPSNKFG), Mr-3 (NVIQAPAQSVAPP NTST), Mr-4 [KENVLNKLKSK(L/I)] and Mr-5 [NAVAAAN(L/I)PG(L/I)V]. None of them contains a disulfide bond. The sequences of conopeptides Mr-2 to Mr-5 do not belong to any category of the known disulfide-poor conopeptides. No significant activity against the above nAChR subtypes were observed for the five conopeptides at 100 µM.

**Conclusion:**

We purified and structurally characterized five novel disulfide-poor conopeptides from *C. marmoreus* crude venom and first investigated their nAChR inhibitory effects. This work expanded our knowledge on the structure and function of disulfide-poor conopeptides from this cone snail venom.

## Background

Cone snails comprise a genus of carnivorous mollusks from the Conidae family that contains more than 700 *Conus* species in total [1-2]. They live in the tropical and subtropical shallow seawater all over the world. These slow-moving mollusks rely on secreting and releasing venom for defense and predation [3-4]. Typically, each cone snail venom contains at least 1000 neuropeptides, called conopeptides or conotoxins, and their composition differs from species to species [4-6]. It is estimated that cone snails can produce up to 1 million different natural peptides. However, less than 0.1% of them has been structurally and functionally characterized to date [7]. Thus, *Conus* venom has been considered an interesting source of peptide-based therapeutics because of their structural and functional diversity and their promising prospects for treating burdensome diseases including neuralgia, addiction, epilepsy, depression, cancer, etc. [8-10]. 

*Conus marmoreus* is a common species in the South China Sea. To date, 176 mature peptide sequences have been recorded in the online database “Conoserver” (http://www.conoserver.org/) [11]. Dutertre *et al.* [12] had identified 105 conopeptide precursor sequences from 13 gene superfamilies from the venom gland transcriptome of *C. marmoreus*, and discovered 2710 and 3172 peptides using MALDI-MS (matrix-assisted laser desorption ionization-mass spectrometry) and ESI-MS (electrospray ionization-mass spectrometry), respectively, from proteomic data of *C. marmoreus* venom*.* Lavergne *et al.* [13] had performed a reanalysis of *C. marmoreus* venom duct transcriptome using algorithm “ConoSorter” and revealed 158 novel conotoxins and 13 new gene superfamilies. These comprehensive transcriptomic and proteomic data showed the vast diversity of the conopeptides from *C. marmoreus* [14]. 

In order to explore novel conopeptides and characterize their structure and function, we prepared and then fractionated the *C. marmoreus* venom. Meticulous purification process was conducted to obtain five novel disulfide-poor conopeptides. Their sequences were identified by integral analysis of MALDI-TOF tandem mass spectrometry data and Edman degradation result. They were named as Mr-1 (DWEYHAHPKPNSFWT), Mr-2 (YPTRAYPSNKFG), Mr-3 (NVIQAPAQSVAPPNTST), Mr-4 [KENVLNKLKSK(L/I)] and Mr-5 [NAVAAAN(L/I)PG(L/I)V]. None of them contains a disulfide bond. In order to investigate their function, these five conopeptides were synthesized by Fmoc-SPPS chemistry, and their inhibitory activities for several nAChR (nicotinic acetylcholine receptors) subtypes (α1β1δε, α3β2, α3β4, α4β2) were investigated. 

## Methods

### Crude venom preparation and peptide isolation

Ten specimens of *C. marmoreus* were collected from shallow sea near Sansha City in South China and were frozen at −80 °C. The crude venom preparation and fractionation processes were conducted as previously described [15]. Briefly, the venom duct of the snail samples were dissected and then extracted by 60% acetonitrile aqueous solution to obtain crude venom powder. The venom powder was dissolved, fractionated by a preparative Waters HPLC e2535 separations module system equipped with a reverse-phase C_18_ column (Vydac Grace, 10 μm, 22 mm × 250 mm, 10 mL/min), and purified by preparative HPLC e2695 system with a reverse-phase C_18_ column (Vydac Grace, 5 μm, 4.6 mm × 250 mm, 0.8 mL/min). Solution A (0.1% TFA in ddH_2_O) and solution B (0.1% TFA in 90% acetonitrile aqueous solution) were used as the mobile phase. The monitoring wavelength was set at 214 nm throughout the fractionation and isolation process. The crude venom was fractionated to obtain 20 fractions named Mar-1−Mar-20 with isocratic elution of 5%−60% solution B in 60 min. Fraction Mar-4 was washed by 30% solution B and subjected to a 25 min isocratic elution of 10%−28% solution B to obtain Mr-2 at 9.86 min and Mr-3 at 10.92 min. Fraction Mar-9 eluted at 40% solution B was separated by a linear gradient of 18%−35% solution B in 20 min to gain Mr-4 at 12.3 min. Mr-5 was yielded at 14.82 min by gradient eluting program of 18%−35% solution B in 20 min from Mar-19, which was washed by 50% solution B. Mr-1 was obtained at 13.65 min by a 20 min gradient program of 23%−38% solution B from the 45% solution B eluted fraction Mar-15. 

The purified conopeptides were subjected to LC-MS (Waters, Acquity I-Class/Xevo UPLC-ESI-TQD-MS, USA) analysis with a C_18_ column (Acquity UPLC Peptide BEH, 130 Å, 1.7 μm, 2.1 mm × 100 mm) with solution A (0.1% formic acid in ddH_2_O) and solution B (0.1% formic acid in acetonitrile) as mobile phase. The detection range of m/z ratio was set at 400−1500. The cone voltage and capillary voltage were 30 V and 3.5 kV, respectively. The desolvation temperature was 550 °C, and the ion source temperature was 150 °C. The gradient program of UPLC analysis was 5%~60% solution B for 6 min, and the flow rate was 0.5 mL/min.

### Peptide sequencing by MALDI-TOF-MS/MS analysis and Edman degradation

The purified conotoxins were all subjected to total reduction by DTT (dithiothreitol) before sequencing. Each conopeptides (0.1 mM in 20% acetonitrile aqueous solution) was mixed with 200 mM DTT (in 20% acetonitrile aqueous solution). The mixtures were reacted at room temperature (25 °C) on a shaker for about 2 hours. Each reaction process was monitored by LC-MS every half an hour. Once the totally-reduced peptides were detected, the reaction solutions were separately purified by getting through a reverse-phase C_18_ column (Vydac Grace, 300 Å, 5 μm, 4.6 mm × 250 mm) to collect the reduced peptides for the follow-up sequence identification. 

Mass spectrometry detection was carried out on a MALDI-TOF-MS/MS spectrometer (Bruker, Ultraflextreme, Germany). The mass axis was calibrated by a peptide mixture (Peptide Calibration Standards II, P/N 8222570, 700−4000 Da). For sample preparation, 1 μL of each reduced conotoxin solution (dissolved in 50% acetonitrile) and 1 μL of HCCA solution (1 mg in 250 μL standard solution) were successively dropped onto the target plate and then dried off. The laser frequency was 1000 Hz. The voltage was set at 19 kV. FlexControl was used to acquire the primary and secondary mass spectra, and flexAnalysis was applied to dispose the data and gain a series of *b* and *y* ions. For peptide sequence identification, Mascot search was performed with the online NCBI or Swiss-prot database. 

The Edman degradation sequencing was performed in a PPSQ-53A Protein sequencer (Shimadzu, Japan). Each reduced peptide was dissolved by 20 μL ddH_2_O. Ten microliter of the testing solution was added to the PVDF (polyvinylidene fluoride) film to dry off, and then the film was transferred to the reactor. A certain PTH (phenylthiohydantoin)-amino acid was yielded after the N-terminal amino acid degradation in each cycle. HPLC analysis of the PTH-amino acid derived from each cycle was detected using a Wakopak Wakosil-PTH-II column (wako, S-PSQ, 4.6 mm× 250 mm) with 40% acetonitrile (< 2.5% acetic acid) as mobile phase. The retention time (*t*_*R*_ ) of the certain PTH-amino acid yielded in each cycle was compared with the *t*_*R*_ of the standards to identify the amino acid until the intact sequence was obtained.

### Peptide synthesis

According to the identified sequences, the five conopeptides were synthesized on a polypeptide synthesis reactor (Shanghai Aladdin Biochemical Technology Co., LTD) by a stepwise solid-phase method using Fmoc (*N*-9-flurenylmethoxycarbonyl) chemistry. The 2-Cl(Trt)-Cl Resin (Tianjin Nankai University Resin Co., LTD) and the Fmoc-L-amino acids [Cishi Biotechnology (Shanghai) Co., LTD] were used. Ninhydrin chromogenic method was applied to detect the Fmoc group on the resin. The synthesized peptide was released from the resin by a mixture of TFA/phenol/thioanisole/water 90/7.5/2.5/5 on a shaker for 0.5−2 hours in the dark. The resin was then removed by filtration and rinsed with TFA three times. The obtained filtrate was added drop by drop into cold diethyl ether (4 °C) to precipitate the peptide. After centrifugation, the crude peptide was subjected to preparative reverse-phase HPLC for purification with gradient elution of 10−35% solution B. The homogeneity of retention time, molecular weight and sequence between the synthesized and the isolated native peptides was individually confirmed by analytical HPLC and MALDI-TOF-MS/MS. 

### Electrophysiological measurements for nAChR blockage

Rat α1, α3, α4, β1, β2, β4, δ and ε nAChR subunit clones were kindly provided by Utah University (Salt Lake City, Utah, USA). *In vitro* cRNA synthesis was conducted as previously reported [16]. *Xenopus laevis* were purchased from Nasco (Fort Atkinson, WI). Mature female *X. Laevis* frogs were anesthetized on ice and dissected for the oocytes, which were subjected to enzymolysis (25 °C, 40 min) by 20 mg trypsin in 40 mL OR-2 buffer (82.5 mM NaCl, 2.0 mM KCl, 1.0 mM MgCl_2_·6H_2_O, 5 mM HEPES, pH 7.5) to obtain individual oocyte. Each subunit cRNA (10−20 ng in 46−59 nL of water) were individually injected into the oocytes to obtain several nAChR subtypes (α4β2, α3β2, α1β1δε, α3β4). α1β1δε cRNA was formed by mixing α1, β1, δ, ε subunits at 2:1:1:1 ratio. α1 and β1 subunit cRNAs were separately mixed at 4:2, 3:2 and 3:4 ratios to obtain α4β2, α3β2 and α3β4 cRNAs. All oocytes were then incubated at 17 °C in ND96 buffer (96.0 mM NaCl, 2.0 mM KCl, 1.8 mM CaCl_2_, 1.0 mM MgCl_2_, 5 mM HEPES, at pH 7.1−7.5) supplemented with 50 mg/L gentamicin for 2−5 days. For electrophysiological measurements, ACh was used to obtain a control response before incubating with conopeptides Mr-1−Mr-5. The control response by ACh was measured under a two electrode voltage clamp amplifier (Axon 900A, Molecular Devices MD, Sunnyvale, CA, USA), at a holding potential of −70 mV. An amount of 10 µM ACh was applied for α1β1δε subtype, and 100 µM ACh was for rat α4β2, α3β2, α3β4 subtypes. Micropipettes were filled with 3 M KCl and had resistances of 0.5−2 MΩ. The elicited current responses were recorded and analyzed using pClamp10 software (MD, Sunnyvale, CA), filtered at 10 Hz, and digitized at 200 Hz. All tested conopeptides (5 µL of 1 mmol/L in ND96) were separately added into the 50 µL cell chamber and incubated for 5 min. The activities of Mr-1−Mr-5 at the heterologously expressed rat nAChRs were determined by comparing the ACh-induced current response after a 5 min incubation with Mr-1−Mr-5 to the average ACh-induced responses before the incubation. α-conotoxin GID (IRDECCSNPACRVNNOHVC) was used as a positive control for blocking α4β2 nAChR. All data were presented as mean ± SEM of 4−9 oocytes.

## 3. Results

### Peptide isolation and sequence identification

**Figure 1.**
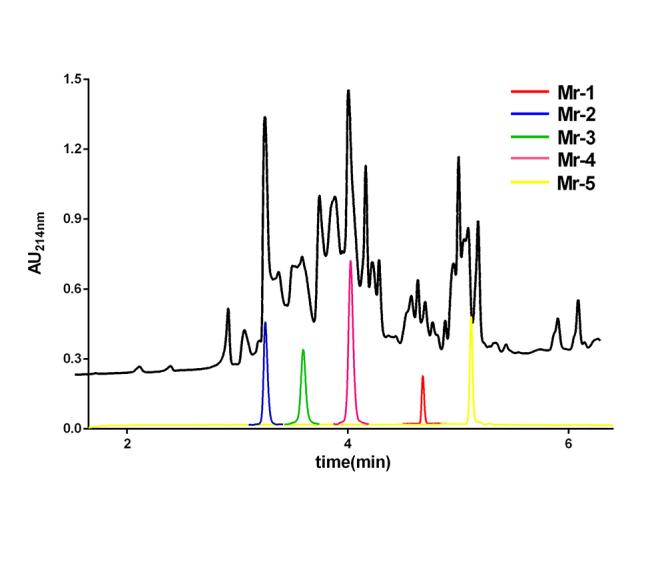
UPLC profiles of *C. marmoreus* venom and the purified conopeptides. The black curve indicates the UPLC profile of crude venom. The red, blue, green, pink and yellow curves represent the UPLC profiles of the purified Mr-1 to Mr-5, respectively.

**Figure 2.**
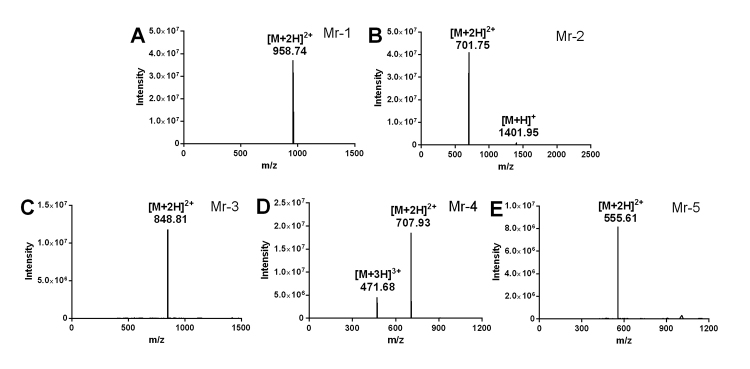
MS spectra of conopeptides Mr-1 to Mr-5. **(A)** Peak at m/z 958.74 [M+2H]^2+^ showed that the molecular weight of Mr-1 was 1915.48 Da. **(B)** Peaks at m/z 701.75 [M+2H]^2+^ and 1401.95 [M+H]^+^ indicated that the molecular weight of Mr-2 was 1400.95 Da. **(C)** 1695.62 Da for Mr-3 was deduced from the peak at 848.81 [M+2H]^2+^. **(D)** Peaks at m/z 471.68 [M+3H]^3+^ and 707.93 [M+2H]^2+^ revealed a molecular weight of 1413.86 Da for Mr-4. **(E)** 1108.95 Da for Mr-5 was confirmed by signal at m/z 555.21 [M+2H]^2+^.

For the venom extraction, 9 mg venom powder of *C. marmoreus* was obtained and subjected to the systematic separation, which led to the isolation of five novel disulfide-poor conopeptides named Mr-1−Mr-5. Their HPLC peaks were illustrated in Figure 1. The five purified conopeptides were individually reduced by DTT. The molecular weights of both the intact peptides (shown in Figure 2) and their reduced forms were respectively determined by UPLC-ESI-TQD-MS (Table 1), which indicated that none of them contains disulfide bonds. Thus, the isolated native peptides were subjected to MALDI-TOF-MS/MS detection and Edman degradation experiment. Their sequences were assigned by comprehensive analysis of mass spectrum and Edman degradation data. In the MALDI-TOF-MS/MS spectrum, b/y ions were generated by CID (collision-induced dissociation) fragmentation at peptide bond, while a/x ions were produced by breaking the Cα−C=O bond. The sequencing results were listed in Table 1. 

**Table 1.** Sequences, molecular weights and numbers of disulfide bonds of the purified conopeptides.

Name	Sequence	Molecular weights before and after reduction	Number of disulfide bond
Mr-1	DWEYHAHPKPNSFWT	1915.47/1915.04	0
Mr-2	YPTRAYPSNKFG	1400.72/1400.94	0
Mr-3	NVIQAPAQSVAPPNTST	1695.62/1695.76	0
Mr-4	KENVLNKLKSK(L/I)	1413.86/1413.88	0
Mr-5	NAVAAAN(L/I)PG(L/I)V	1108.95/1108.58	0

**Figure 3.**
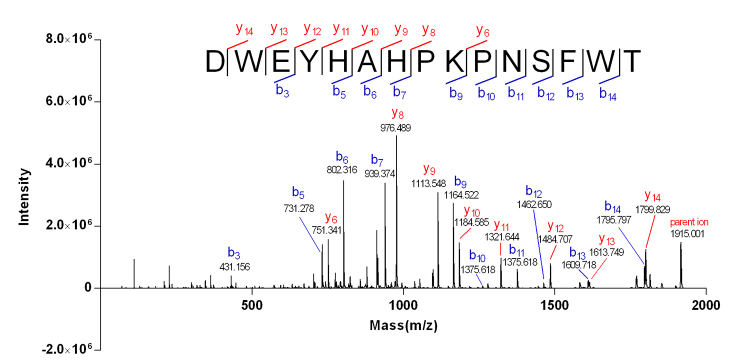
MALDI-TOF-MS/MS spectrum of Mr-1. The primary sequence of Mr-1 was determined by the consecutive b/y ions generated from CID fragmentation.

Peak at m/z 958.74 [M+2H]2+ observed in the ESI-TQD-MS spectrum (Figure 2A) coincided with the parent ion peak at m/z 1915.001 in the MALDI-TOF-MS/MS spectrum (Figure 3). The Edman degradation sequencing (Additional file 1) of Mr-1 illustrated the sequence to be DWEYHAHPKPNSFWT. Two fragments of DWEYHAH and PNSFWT were deduced from the consecutive *b* ions (*b*_5_−*b*_7_, *b*_9_−*b*_14_) and *y* ions (*y*_*6*_ *, y*_8_−*y*_14_) in the MS/MS spectrum, which was compatible with the Edman degradation result. Hence, the primary sequence of Mr-1 was undoubtedly identified to be DWEYHAHPKPNSFWT, which was a novel disulfide-poor conomarphin peptide. Through sequence alignment, Mr-1 was found to be similar to peptide conomarphin-Mr1 (DWEYHAHPKONSfWT, O: hydroxyproline, f: D-phenylalanine), which was previously purified from *C. marmoreus* venom [17]. Thus, Mr-1 can be named as conomarphin-Mr3.

**Figure 4.**
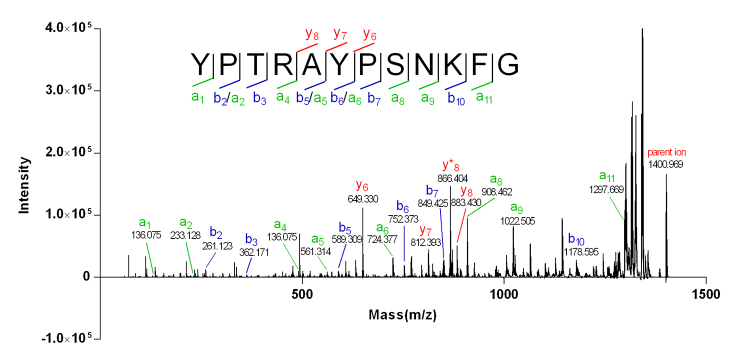
MALDI-TOF-MS/MS spectrum of Mr-2. Serial *b*/*y* ions and *a* ions generated from CID fragmentation confirmed the primary sequence of Mr-2.

The successive *b* ions (*b*_2_−*b*_3_, *b*_5_−*b*_7_), *a* ions (*a*_1_−*a*_2_, *a*_4_−*a*_6_, *a*_8_, *a*_9_, *a*_11_) and *y* ions (*y*_6_−*y*_8_) observed in the MALDI-TOF-MS/MS spectrum (Figure 4) revealed a YPTRAYPSNKF fragment in the sequence of Mr-2. The detection of *b*_10_ ion (m/z 1178.5953) and *a*_11_ ion (m/z 1297.6688) indicated a phenylalanine (Phe, F, 147.1739) residue at position 11. For the assignment of residue at the *C*-terminal of Mr-2, a glycine residue (57.0513) could be easily speculated from the mass difference (57.5097) between the parent ion (m/z 1400.969) and fragment YPTRAYPSNKF (m/z 1343.4863). Thus, the primary sequence of Mr-2 was determined to be YPTRAYPSNKFG, which was perfectly consistent with the Edman degradation sequencing result (YPTRAYPSNKFG, Additional file 2).

**Figure 5.**
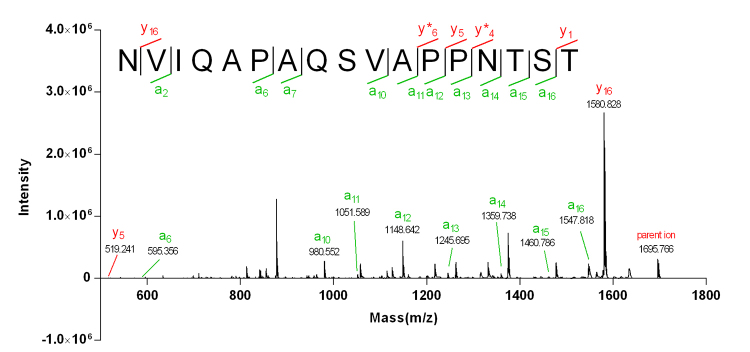
MALDI-TOF-MS/MS spectrum of Mr-3. The observation of *y* and *a* ions help confirming the partial sequence of Mr-3.

The molecular weight of Mr-3 was determined to be 1695.62 Da based on the observation of peak at m/z 848.81 [M+2H]^2+^ in the ESI-MS spectrum (Figure 2C), which was in accordance with the parent ion (m/z 1695.766) presented in MALDI-TOF-MS/MS spectrum (Figure 5). A doubtless fragment sequence of NVIQAPAQSVAP*N*** in Mr-3 was confirmed by the Edman degradation sequencing (Additional file 1). The series of *a* ions (*a*_10_−*a*_*16*_ ) observed in MALDI-TOF-MS/MS spectrum illustrated that the residues at positions 10−16 were VAPPNTS. Based on the mass difference (101.1) between NVIQAPAQSVAPPNTS (m/z 1593.737) and the parent ion mass (m/z 1695.766), a threonine (Thr, m/z 101.10392) residue was deduced at the *C*-terminus (position 17). Thus, the intact sequence of Mr-3 was assigned as NVIQAPAQSVAPPNTST.

**Figure 6.**
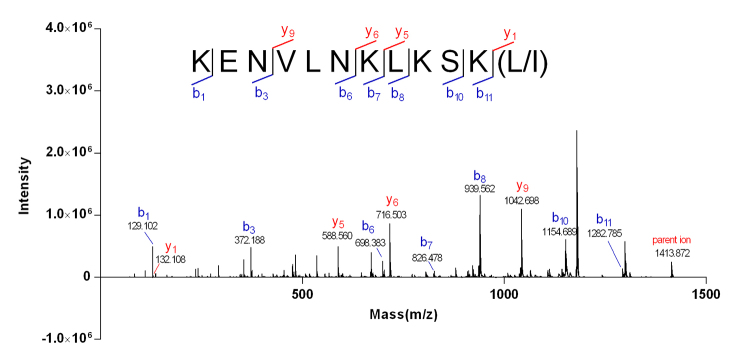
MALDI-TOF-MS/MS spectrum of Mr-4. Partial sequence of Mr-4 was assigned by the observed *b*/*y* ions, corresponding with the Edman degradation sequencing. L/I at position 12 could not be distinguished by MS/MS analysis.

Peak at m/z 707.93 [M+2H]^2+^ in the MS spectrum (Figure 2D) of Mr-4 indicated the peptide mass to be 1413.86 Da, which was consistent with the parent ion (m/z 1413.872) in MALDI-TOF-MS/MS spectrum (Figure 6). The Edman degradation sequencing of Mr-4 showed an unambiguous sequence fragment of KENVLNKLKS** (Additional file 4). The observation of successive *b* ions (*b*_6_−*b*_8_) and few *y* ions (*y*_5_, y_6_, *y*_9_) were also detected in MS/MS spectrum to verify the Edman degradation data. KENVLNKLKS** confirmed the residues at positions 5 and 8 to be Leu (L), which could not be identified by MS/MS sequencing. The *y*_1_ (m/z 132.108) and *b*_11_ (m/z 1282.758) ions revealed the residues at positions 11 and 12 should be lysine (K) and L/I, respectively. MS/MS data could not distinguish between Leu and Ile residues. Thus, Mr-4 was a novel 12-residue peptide whose complete sequence was KENVLNKLKSK(L/I).

**Figure 7.**
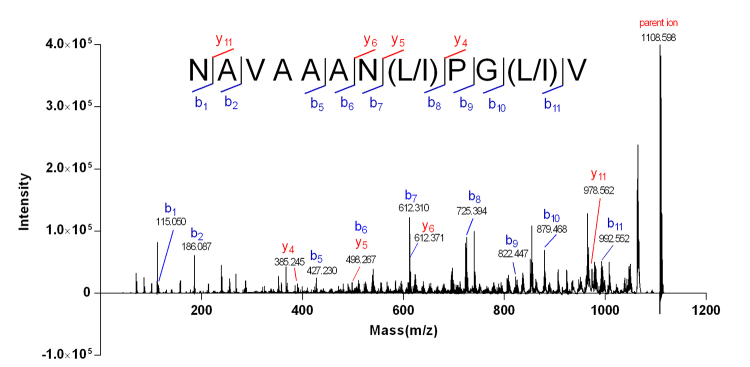
MALDI-TOF-MS/MS spectrum of Mr-5. Partial sequence of Mr-5 was ascertained by the *b*/*y* ions generated from CID fragmentation. L/I at positions 8 and 11 could not be distinguished by MS/MS analysis.

Similarly, the sequential *b* ions (*b*_5_−*b*_11_) and *y* ions (*y*_4_−*y*_6_) recorded in the MALDI-TOF-MS/MS spectrum (Figure 7) revealed the fragment AN(L/I)PG(L/I)V at positions 6−12 of Mr-5. The fragment NAVA**N***** was speculated from Edman degradation sequencing (Additional file 5). The result combined with the observation of the series of *b* ions (*b*_1_−*b*_2_ and *b*_5_) showed that the residues at positions 1−5 were NAVAA. Therefore, Mr-5 was ascertained to be NAVAAAN(L/I)PG(L/I)V.

So far, 176 mature peptides have been reported from *C. marmoreus*, 21 of them are disulfide-poor conopeptides (Table 2). Except for conomarphins and contryphan-M, the rest of them were deduced from transcriptomic and proteomic data. Their conopeptide class and pharmacological activity have not been identified yet. 

**Table 2.** Disulfide-poor conopeptides discovered from *C. marmoreus* species.

Name	Sequence	Conopeptide class	Target	Reference
Conomarphin-Mr1	DWEYHAHPKONSfWT	Conomarphin	No data	[12, 17]
Conomarphin-Mr2	DWVNHAHOQONSIWS	Conomarphin	No data	[12]
Conomarphin-14	DWEYHAHPKONSfW	Conomarphin	No data	[12, 18]
Conomarphin-8	HPKONSfW	Conomarphin	No data	[12, 18]
Contryphan-M	NγSγCPwHPWC#	Contryphan	No data	[12,19]
contryphan-M2	ESECPWHPWC#	Contryphan	No data	[12]
Mr034	DCCPVAGMPLWMQPLLWMTSFVIGTSSSNE	Unclassified	No data	[12]
Mr035	LVVGDQLCYRVLIKCLMNK	Unclassified	No data	[12]
Mr036	TLQNASEQTLLPRLGIVLRV	Unclassified	No data	[12]
Mr038	NγFLTHTFS(Btr)HPTWCPWC#	Unclassified	No data	[12]
Mr080	STIPSLGSEWDDGW	Unclassified	No data	[12]
Mr081	TLQMLGTNAAAQAGNCAASGMMGGKGK	Unclassified	No data	[12]
Mr082	TLQMLRTNAAAQAGNCAASGMMGGKGK	Unclassified	No data	[12]
Mr083	QMLRTNAAAQAGNCAASGMMGGKENDLR	Unclassified	No data	[12]
Mr086	TLTNASEQTLLPRLGIVLRVG	Unclassified	No data	[12]
Mr087	TLQKLLNKTLLPNSATVL	Unclassified	No data	[12]
Mr088	TLTKAFEQTLLPNSATVL	Unclassified	No data	[12]
Mr103	GCGMMRVTVQQPLSPEALSWTPNCNVS	Unclassified	No data	[12]
Mr105	AMVIDGQKLMHDCAIANDYIDDPWWTLNLGAFEEKRVYHSMLSELVFCLNAFLQ	Unclassified	No data	[12]
Mr106	CIGSCDSTVWHRV	Unclassified	No data	[12]
Mr107	DVKSIGSWDFTVWHRV	Unclassified	No data	[12]
Mr-1/conomarphin-Mr3	DWEYHAHPKPNSFWT	Conomarphin	Unknown	This work
Mr-2	YPTRAYPSNKFG	Unclassified	Unknown	This work
Mr-3	NVIQAPAQSVAPPNTST	Unclassified	Unknown	This work
Mr-4	KENVLNKLKSK(L/I)	Unclassified	Unknown	This work
Mr-5	NAVAAAN(L/I)PG(L/I)V	Unclassified	Unknown	This work

O: 4-hydroxyproline; f: D-phenylalanine; γ: γ-carboxylic glutamic acid; w: D-tryptophan; #: C-term amidation; Btr: bromotryptophan.

### Biological effect on nAChRs

**Figure 8.**
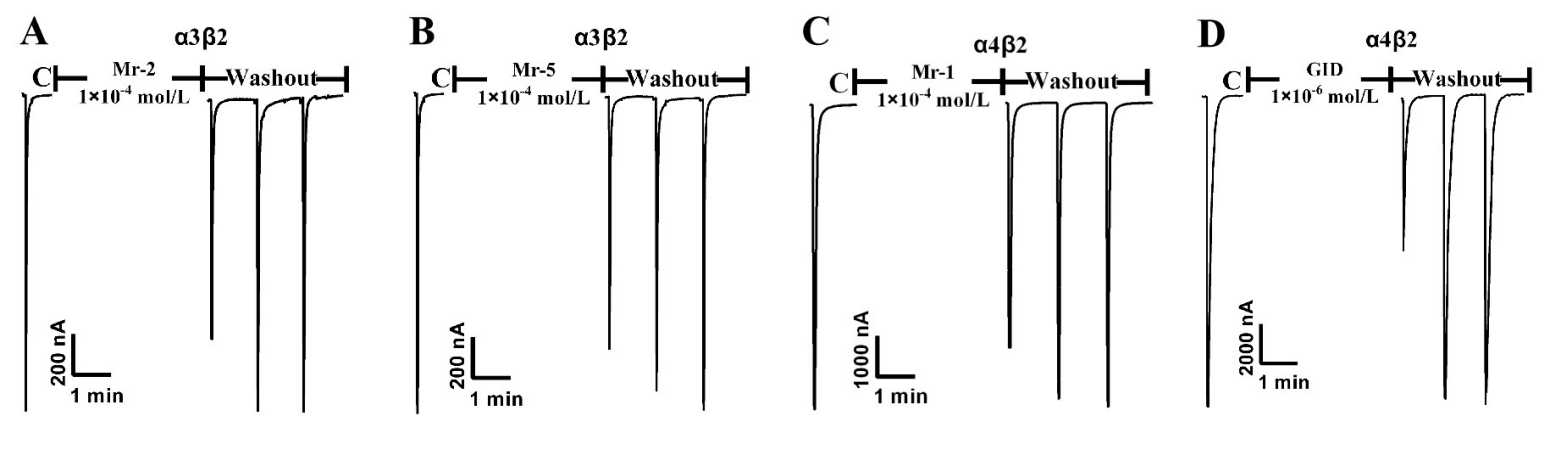
Biological effect on nAChRs of Mr-1, Mr-2 and Mr-5. Current trace of inhibition of α3β2 nAChR by **(A)** Mr-2 and **(B)** Mr-5. Current trace of inhibition of α4β2 nAChR by **(C)** Mr-1 and **(D)** GID. GID was used as a positive antagonist control for α4β2 nAChR.

In order to investigate the nAChR-associated activity of the five disulfide-poor conopeptides (Mr-1−Mr-5), they were synthesized according to the identified sequences. Since Ile and Leu are isomers whose hydrophobicity, dispersion and ionic properties are quite similar, we chose Leu to substitute I/L in the sequences of Mr-4 and Mr-5. Mr-4 and Mr-5 were synthesized as KENVLNKLKSKL and NAVAAANLPGLV, respectively. If the sequences show certain activity, the sequences contained Ile would be synthesized for further investigation. Several rat nAChR subtypes (α4β2, α3β2, α1β1δε, α3β4) were expressed in the *X. Laevis* oocytes, and subjected to electrophysiological experiments (Figure 8). Both Mr-2 and Mr-5 separately showed about 30 ± 6.5 % of ACh-evoked currents mediated by α3β2 nAChR (Figure 8A and 8B), while Mr-1 inhibited 20 ± 8.5 % of α4β2 nAChR ACh-evoked currents (Figure 8C), at the concentration of 100 µM. On the other hand, 1 µM GID inhibited 55 ± 4.3 % of α4β2 nAChR ACh currents (Figure 8D). No activity was observed against α3β4 and α1β1δε nAChRs for all the five conopeptides testing at 100 µM. Thus, all the five novel conopeptides presented no significant activity against the above nAChRs.

## Discussion

nAChRs are a class of trans-membrane ligand-gated ion channel receptors and have been identified as targets for various diseases such as pain, addiction, depression and cancer, etc [8, 20-21]. The α4β2 subtype, a neurotype nAChR, has been proven to be a crucial target associated with addiction [22]. For the weaker withdrawal reaction and less relapsing rate, α4β2 nAChR antagonist have become an research hot spot on searching potential candidates for smoking cessation drug [22-24]. Several smoking cessation drugs such as bupropion and varenicline, have launched into the market for years [25-26]. However, most of them have ended up with unsatisfactory therapeutic effects due to the low selectivity and potency for α4β2 nAChR [27-29]. The main reason for the low selectivity is that α4β2 nAChR antagonists (such as GIC and GID) also show potent inhibitory activity on α3β2 nAChR [30-31]. Thus, we had investigated the α4β2 and α3β2 nAChRs inhibitory effects of the *C. marmoreus* venom fractions. Several fractions had been shown with certain α4β2/α3β2 nAChRs selectivity [15], which indicated *C. marmoreus* venom as a good natural source for discovering novel α4β2 nAChR antagonist with low selectivity against α3β2 nAChR. In this work, we purified the venom fractions of *C. marmoreus* collected from the South China Sea and structurely characterized and functionally identified five novel disulfide-poor conopeptides. Their sequences were assigned by comprehensive analysis of MALDI-TOF tandem mass data and Edman degradation sequencing. They were named Mr-1 (DWEYHAHPKPNSFWT), Mr-2 (YPTRAYPSNKFG), Mr-3 (NVIQAPAQSVAPPNTST), Mr-4 [KENVLNKLKSK(L/I)] and Mr-5 [NAVAAAN(L/I)PG(L/I)V]. Mr-1 is structurely similar to conomarphin-Mr1, which have been discovered from *C. marmoreus* venom since 2008. No activity study have been recorded for conomarphin-Mr1. Mr-1−Mr-5 were first reported and synthesized by Fmoc-SPPS chemistry, and their activity at several nAChR subtypes (α1β1δε, α3β2, α3β4, α4β2) were investigated. All the five conopeptides showed no significant activity against the above nAChR subtypes. α1β1δε nAChR, a muscular type nAChR, is associated with the muscle contraction and is considered as a target contributing to the venom toxicity for cone snail predation. Mr-1−Mr-5 showed no activity against α1β1δε nAChR, which meant that they do not affect the muscle contraction. 

Generally, disulfide-rich conopeptides, conotoxins, are considered to be the dominant component of the *Conus* venom and preferentially present the neuroactive pharmacology [8, 32]. Although disulfide-poor conopeptides occupy a minor portion of *Conus* venom, they still have caught interest for intensive investigation [33]. Disulfide-poor conopeptides can be divided into many subgroups, including contulakins, conantokins, conorfamides, conolysins, conopressins, contryphans, conophans, conomarphins, conomaps, conoCAPs, conoNPYs, conoGAYs and hormone-like conopeptides, which have been found to interact with diverse targets (such as ion channels, vasopressin receptor, NMDA receptor, neurotensin receptor) and have shown potential prospects as drug candidates for cardiovascular disease, epilepsy, mood control and pain release [34]. To date, two of them (contulakin-G and conantokin-G) have already reached clinical trials for alleviating pain, which indicates disulfide-poor conopeptides as promising leads for drug discovery [35-36]. 

Mr-1, which was identified as a conomarphin, does not present the common post-translational modification (PTM) as previous conomarphins. The hydroxylation of 10-Pro residue and the epimerization of Phe-13 residue in Mr-1 were absent, suggesting the maturation of conomarphins could be accomplished without the above PTMs. As for Mr-2−Mr-5, they differ from each other and do not belong to any category of the known disulfide-poor conopeptides. Their conopeptide class could not be ascertained until their pharmacological targets are identified.

## Conclusion

We purified and structurally characterized five novel disulfide-poor conopeptides (Mr-1 to Mr-5) from *C. marmoreus* crude venom and investigated their activity at the rat nAChRs. This work expanded our knowledge on the structure and function of disulfide-poor conopeptides from *C. marmoreus* venom, which provided new information for their further exploring. Their activities against other types of receptors (such as ion channels, vasopressin receptor, NMDA receptor, neurotensin receptor) and effects on animal models remain to be further studied. 

### Abbreviations

CID: collision-induced dissociation; ddH_2_O: double distilled water; ESI: electrospray ionization; HCCA: cyano-4-hydroxycinnamic acid; HEPES: 4-(2-hydroxyethyl)-1-piperazineethane-sulfonic acid; HPLC: high-performance liquid chromatography; LC-MS: liquid chromatography-mass spectrometry; MALDI-TOF: matrix-assisted laser desorption ionization-time of flight; MS: mass spectrometry; N-methyl-D-aspartic acid receptor; Na_2_-EDTA: ethylenediaminetetraacetic acid disodium salt dihydrate; nAChRs: nicotinic acetylcholine receptors; NET: norepinephrine transporter; NMDA: PTH: phenylthiohydantoin; PTM: post-translational modification; TCEP: tris-(2-carboxyethyl)-phosphine; TFA: trifluoroacetic acid; TQD: triple quadrupole; UPLC: ultra-performance liquid chromatography; VGCC: voltage-gated calcium channel; VGPC: voltage-gated potassium channel. 

## Supplementary material

The following online material is available for this article:

Additional file 1. HPLC peak area data of amino acids in Edman degradation cycle of Mr-1.

Additional file 2. HPLC peak area data of amino acids in Edman degradation cycle of Mr-2.

Additional file 3. HPLC peak area data of amino acids in Edman degradation cycle of Mr-3.

Additional file 4. HPLC peak area data of amino acids in Edman degradation cycle of Mr-4.

Additional file 5. HPLC peak area data of amino acids in Edman degradation cycle of Mr-5.
