# Supplementary material for: Isolation and characterization of five novel disulfide-poor conopeptides from Conus marmoreus venom
Source: J Venom Anim Toxins Incl Trop Dis. 2022 May 18;28:e20210116. doi: 10.1590/1678-9199-JVATITD-2021-0116 (PMC9136937; doi:10.1590/1678-9199-JVATITD-2021-0116)
Supplement: Additional file 1. [file 1678-9199-jvatitd-28-e20210116-s1.pdf]

J Venom Anim Toxins incl Trop Dis
https://doi.org/10.1590/1678-9199-JVATITD-2021-0116

Supplementary Material to "Isolation and characterization of five novel disulfide-poor conopeptides from *Conus marmoreus* venom"

Additional file 1. HPLC peak area data of amino acids in Edman degradation cycle of Mr-1.

Component	1	2	3	4	5	6	7	8
**Asp**	289.08	0.4	0.67	0.97	0.93	0.96	0.93	0.97
**Glu**	1.04	0.78	583.61	0.18	0.27	0.84	19.22	0.78
**Asn**	0.83	4.37	0.85	0.51	0.88	13.37	566.05	0.32
**Gln**	1	1	42.87	0.78	2.7	15.47	0.81	0.61
**Ser**	22.87	0.64	0.93	0.92	5.67	0.72	0.08	100.8
**Thr**	3.87	0.68	0.9	2.9	0	0.53	28.06	0.79
**His**	0.16	29.99	30.5	154.14	19178.71	0.17	12590.85	0.25
**Gly**	0.73	17.94	6.05	0.96	189.82	187.27	0.29	3.51
**Ala**	0.8	18.62	19.87	139.86	280.5	39165.23	0.21	0.17
**Tyr**	0.48	1.18	486.54	42226.9	0.14	0.07	0.18	0.36
**Arg**	0.03	88.91	0.14	0.25	0.44	6.66	0.76	11.37
**Met**	0.44	0.68	0.24	0.91	0.63	0.84	0	0.94
**Val**	6.89	0.74	2.96	1.16	0.93	9.76	0.81	0.76
**Pro**	7.55	0.72	17.13	25.16	35.31	22.6	247.48	18002.47
**Trp**	0.01	46765.49	0.08	0.07	0.24	0.5	0.85	0.83
**Phe**	0.08	129.1	77.51	111.08	92.14	98.37	103.37	113.7
**Lys**	0.87	0.97	0.74	18.69	24.62	28.99	17.95	211.87
**Ile**	0.75	3.62	0.73	0.96	0.84	0.93	0.16	0.63
**Leu**	0.73	6.13	1.25	5.31	2.44	5.57	0.75	5.89

Component	9	10	11	12	13	14	15
**Asp**	0.98	3.72	269.94	0.67	0.7	0.78	0.88
**Glu**	9.45	22.14	92.66	0.76	0.55	0.51	0.6
**Asn**	0.43	42.5	6094.57	0.5	0.33	0.3	0.34
**Gln**	41.08	2.17	0.29	41.99	23.14	11.93	13.74
**Ser**	0.45	0.62	8.54	1436.82	0.56	0.36	0.34
**Thr**	30.81	19.16	0.55	0.19	0.65	0.76	8.19
**His**	0.27	0.18	0.5	0.64	0.75	0.82	0.86
**Gly**	1.63	2.29	0.89	0.74	0.91	2.07	0.93
**Ala**	0.2	0.3	0.68	8.33	0.78	0.74	0.89
**Tyr**	0.77	0.98	0.78	0.91	0.88	0.95	0.9
**Arg**	0.57	0.19	0.82	58.75	0.64	0.33	0.26
**Met**	1.36	0	1.61	1.2	0.75	0.99	0.45
**Val**	14.93	0.73	0.87	2.3	0.85	0.85	1.02
**Pro**	0.36	3200.96	0.39	0.29	0.32	0.42	0.5
**Trp**	0.78	0.67	0.75	1.05	0.78	23.73	0.75
**Phe**	0.72	0.79	0.92	30.92	757.04	0.71	0.47
**Lys**	22335.08	0.31	0.2	0.19	0.21	0.39	0.54
**Leu**	6.25	0.79	0.75	2.25	4.5	2.88	2.3
