# Supplementary material for: Isolation and characterization of five novel disulfide-poor conopeptides from Conus marmoreus venom
Source: J Venom Anim Toxins Incl Trop Dis. 2022 May 18;28:e20210116. doi: 10.1590/1678-9199-JVATITD-2021-0116 (PMC9136937; doi:10.1590/1678-9199-JVATITD-2021-0116)
Supplement: Additional file 2. [file 1678-9199-jvatitd-28-e20210116-s2.pdf]

J Venom Anim Toxins incl Trop Dis
https://doi.org/10.1590/1678-9199-JVATITD-2021-0116

Supplementary Material to "Isolation and characterization of five novel disulfide-poor conopeptides from *Conus marmoreus* venom"

Additional file 2. HPLC peak area data of amino acids in Edman degradation cycle of Mr-2.

Component	1	2	3	4	5	6
Asp	55.63	0.68	2902.13	0.39	0.38	0.63
Glu	33.31	0.48	29.42	635.45	0.75	2497.17
Asn	0.02	555.71	418.19	0.76	0.84	0.82
Gln	0.98	0.39	133.46	65.19	0.79	0.85
Ser	146.86	0.25	0.86	7.29	2.77	33.06
Thr	0.85	24.49	77519.85	0.49	0.32	0.25
His	6.06	7.77	2996.48	0.34	0.28	0.28
Gly	16.2	0.73	0.49	10.31	0.03	0.49
Ala	0.59	18.58	159.18	857.91	68437.66	0.6
Tyr	146096.5	0.04	0.15	0.02	0.89	115160.9
Arg	0.67	37.4	115.52	28819.35	0.48	0.32
Met	20.14	10.14	3.15	1.96	0	0
Val	6.05	0.92	0.99	8.8	0.66	0.94
Pro	0	114738.9	0.32	0.29	0.29	0.33
Trp	32.3	0.05	0.55	0.23	12.83	0
Phe	8.91	0.68	4.93	24.57	0.48	0.59
Lys	36.43	0.27	260.42	0.4	0.92	0.8
Ile	0.89	1.79	0.93	1.11	0.9	0.62
Leu	0.62	8.42	0.49	0.8	2.79	1.37
**Component**	**7**	**8**	**9**	**10**	**11**	**12**
Asp	0.57	581.96	0.71	0.59	0.53	0.79
Glu	0.3	606.08	0.09	0.81	0.13	0.66
Asn	0.61	85.02	3342.44	0.47	0.2	0.28
Gln	0.81	527.41	0.4	0.44	0.74	0.89
Ser	62.42	15444.14	0.32	0.32	0.15	0.25
His	0.18	0.41	0.63	0.65	0.54	0.38
Gly	0.82	14.07	0.76	22.01	0.77	14.74
Ala	0.26	0.38	0.17	0.29	0.19	0.63
Tyr	0.4	0.43	0.17	0.26	0.19	0.3
Arg	0.15	490.39	0.21	0.34	0.17	0.28
Met	0	0.82	0.58	1.62	0.43	0.56
Val	0.96	8.2	0.71	0.72	0.94	0.96
Pro	33600.39	58372.44	0.19	0.3	0.16	0.31
Trp	0	10.56	0.46	0.55	0.64	0.6
Phe	1.13	22.87	33.9	0.44	40.97	0.72
Lys	0.45	7.41	0.22	298.65	0.31	0.28
Ile	0.37	0.94	0.77	0.42	0.88	0.88
Leu	0.59	0.74	0.89	1.75	0.75	0.73
