# Supplementary material for: Isolation and characterization of five novel disulfide-poor conopeptides from Conus marmoreus venom
Source: J Venom Anim Toxins Incl Trop Dis. 2022 May 18;28:e20210116. doi: 10.1590/1678-9199-JVATITD-2021-0116 (PMC9136937; doi:10.1590/1678-9199-JVATITD-2021-0116)
Supplement: Additional file 3. [file 1678-9199-jvatitd-28-e20210116-s3.pdf]

J Venom Anim Toxins incl Trop Dis
https://doi.org/10.1590/1678-9199-JVATITD-2021-0116

Supplementary Material to "Isolation and characterization of five novel disulfide-poor conopeptides from *Conus marmoreus* venom"

Additional file 3. HPLC peak area data of amino acids in Edman degradation cycle of Mr-3.

Component	1	2	3	4	5	6	7
Asp	6969.21	0.06	0.47	64.17	0.67	0.83	0.97
Glu	36.05	2405.91	0.08	456.47	0.36	0.41	0.8
Asn	77314.8	0.08	0.06	163.66	0.2	80.7	324.85
Gln	0	282.16	419.64	70552.93	0.36	0.21	0.3
Ser	0.29	636.97	0.64	0	0.79	0.94	90.9
Thr	0.78	13.36	71.87	55.33	0.59	0.93	124.98
His	120.23	0.22	0.87	1.8	0.65	0.31	0.95
Gly	2.16	0.76	0.95	0.92	0.09	0.71	1
Ala	0.56	0.27	642.74	1897.9	98366.73	0.42	47591.9
Tyr	0.63	0.46	0.98	5.25	68.2	0.71	16.51
Arg	0.46	55.07	0.59	12.95	70.47	0.73	13.81
Met	1.07	0.85	0	4.17	0.68	0.38	0.26
Val	0	137301.9	0.13	0.17	0.5	0.6	0.96
Pro	0.12	121.06	0.07	281.15	1204.71	98334.27	0.33
Trp	0.12	23.43	35.43	0.22	19.49	0.19	25.9
Phe	0.75	6.52	0.48	0.83	0.52	0.7	8.68
Lys	0.56	4.71	0	0	5.59	0.28	4.2
Ile	0.19	104.37	120977	0.14	0.17	0.18	0.19
Leu	0.8	6.02	105.37	0.25	0.98	0.94	0.85
**Component**	**8**	**9**	**10**	**11**	**12**	**13**	**14**
Asp	82.28	346.42	108.21	0.64	0.55	0.62	1249.92
Glu	221.24	135.45	0.06	0.21	0.32	0.41	61.53
Asn	0.62	0.48	345.45	250.88	173.85	0.67	25064.04
Gln	67006.36	0.3	0.64	0.23	0.24	0.31	5.09
Ser	0	12205.1	14861.6	0.32	0.21	0.17	0.93
Thr	241.49	0.38	147.33	99	0.71	0.43	161.75
His	0.8	0.79	3.54	0.74	0.58	0.45	1.77
Gly	9.78	0.75	4.36	8.82	0.84	0.88	11.43
Ala	0.56	0.16	0.54	49275.69	0.51	0.19	0.49
Tyr	0.75	0.49	4.33	1.41	0.7	0.7	0.92
Arg	0.71	1474.49	1486.79	0.31	0.26	0.14	2.92
Met	0.38	0.75	18.17	0	6.11	9.96	0.73
Val	0.89	0.54	59155.21	0.55	0.26	0.16	0.58
Pro	0.26	0.14	0.92	2.6	32779.36	0.65	0.99
Trp	0.66	0.75	0.65	2.67	0.33	0.51	0.95
Phe	0.6	29.38	148.48	0.49	0.11	2.56	1.27
Lys	3.03	5.43	0.57	0.71	0.52	0.33	0.87
Ile	0.42	0.41	11.3	0.56	0.56	0.54	0.85
Leu	1	0.63	0.94	3.95	0.81	0.81	3.43
