# Supplementary material for: Isolation and characterization of five novel disulfide-poor conopeptides from Conus marmoreus venom
Source: J Venom Anim Toxins Incl Trop Dis. 2022 May 18;28:e20210116. doi: 10.1590/1678-9199-JVATITD-2021-0116 (PMC9136937; doi:10.1590/1678-9199-JVATITD-2021-0116)
Supplement: Additional file 4. [file 1678-9199-jvatitd-28-e20210116-s4.pdf]

J Venom Anim Toxins incl Trop Dis
https://doi.org/10.1590/1678-9199-JVATITD-2021-0116

Supplementary Material to "Isolation and characterization of five novel disulfide-poor conopeptides from *Conus marmoreus* venom"

Additional file 4. HPLC peak area data of amino acids in Edman degradation cycle of Mr-4.

Component	1	2	3	4	5	6
Asp	0.26	345.05	17438.85	0.03	0.39	8010.02
Glu	0.14	19839.66	0.47	0.07	0.1	0.65
Asn	220.38	0.4	65589.23	0.07	0.09	66629.14
Gln	870.66	0.41	0	641.55	1255.16	0
Ser	37.12	0.7	22.27	0.31	0.44	0.17
Thr	84.72	0.09	0.78	101.16	13.19	23.33
His	6.34	0.48	229.71	0.25	0.39	112.23
Gly	19.12	0.74	0.6	54.82	0.68	0.52
Ala	21.31	0.77	0.91	0.65	17.98	0.47
Tyr	0.26	45.75	0.47	0.92	0.49	0.95
Arg	52.4	0.55	0.76	2197.53	0.04	0.48
Met	2.84	0.04	0.76	0.54	0	0
Val	0.43	317.94	0.32	190870.7	0.05	0.08
Pro	25.75	0.44	0.72	328.78	0.32	0.58
Trp	65.25	0.28	0.25	7.73	0	0.33
Phe	0.44	24.49	0.09	1.56	236.46	74.06
Lys	244072.3	0.04	0.11	0.27	0.65	0.91
Ile	225467.3	0.01	0.5	0.36	0.9	4024.46
Leu	0.38	168.14	373.97	278.92	177510.5	0.04
**Component**	**7**	**8**	**9**	**10**	**11**	**12**
Asp	0.07	0.38	10.4	0.73	0.77	0.99
Glu	0.95	0.76	0.19	165.7	122.01	81.18
Asn	0.08	0.07	0.15	0	13.72	16.89
Gln	414.08	304.06	0.6	0.7	0.71	0.97
Ser	0.34	0.67	5.12	262.98	0.05	0.35
Thr	25.33	9.74	0.13	0.8	0.3	0
His	0.19	1166.44	0.11	0.51	0.22	0.56
Gly	172.03	0.47	42.41	0.43	0.72	4.61
Ala	12026.82	0.05	0.15	0.9	1	0.98
Tyr	8.47	0.14	0.53	0.91	7.76	9.1
Arg	21.88	0.13	0.57	0.55	0.77	0.8
Met	113.04	0.1	1.63	1.9	0.28	0
Val	0.26	0.43	0.51	0.46	0.24	0.93
Pro	0.67	0.47	0.89	0.47	0.73	0
Phe	0.04	41.59	0.19	0.61	15.23	0
Lys	192845.8	0.04	3117.88	0.03	10.97	23.15
Ile	0.34	0.33	0.7	0.35	0.49	15.97
Leu	0.13	29915.23	0.06	0.09	0.19	0.38
