# Supplementary material for: Isolation and characterization of five novel disulfide-poor conopeptides from Conus marmoreus venom
Source: J Venom Anim Toxins Incl Trop Dis. 2022 May 18;28:e20210116. doi: 10.1590/1678-9199-JVATITD-2021-0116 (PMC9136937; doi:10.1590/1678-9199-JVATITD-2021-0116)
Supplement: Additional file 5. [file 1678-9199-jvatitd-28-e20210116-s5.pdf]

J Venom Anim Toxins incl Trop Dis

https://doi.org/10.1590/1678-9199-JVATITD-2021-0116

Supplementary Material to "Isolation and characterization of five novel disulfide-poor conopeptides from *Conus marmoreus* venom"

Additional file 5. HPLC peak area data of amino acids in Edman degradation cycle of Mr-5.

Component	1	2	3	4	5	6
Asp	2089.87	0.08	0.51	0.82	0.85	1
Glu	0.96	0.38	0.21	0.06	0.61	0.91
Asn	34044.87	0.03	0.08	0.24	0.4	0.71
Gln	0.95	8.6	0.76	0.72	0.74	0.86
Ser	15.86	0.16	7.14	0.7	1.97	4.76
Thr	0.87	1.99	0.94	1.06	0.89	0.9
His	28.73	0.25	0.05	0.74	0.56	1.05
Gly	0.72	22.19	25.45	2.4	7.67	1.61
Ala	0	29381.9	0.07	3812.01	0.24	0.28
Tyr	0.52	12.28	0.74	0.85	0.83	0.86
Arg	0.35	14.41	0.1	1.59	0.8	0.79
Met	0.72	0.42	1.47	0.05	0.9	0.65
Val	0.64	40.8	16364.26	0.09	0.14	0.25
Pro	0.79	1	13.94	0.48	0.81	1.15
Trp	0.46	0.14	0.47	0.75	0.5	0.61
Phe	0.48	5.94	0.78	0.89	0.89	0.94
Lys	0.19	2.65	0.27	0.26	0	2.35
Ile	0.07	0.51	0.13	0.94	0.79	1.18
Leu	0.6	11.97	6.96	0.78	0.72	0.95
**Component**	**7**	**8**	**9**	**10**	**11**	**12**
Asp	0.95	0.97	0.98	2.59	0.87	0.89
Glu	1.68	0.87	0.86	0.86	0.93	0.87
Asn	27.62	0.49	0.56	0.65	0.67	1.9
Gln	0.89	0.89	0.88	0.93	0.88	0.92
Ser	1.56	2.75	4.25	4.45	3.33	1.32
Thr	0.9	0.91	0.84	0.91	0.88	0.91
His	0.53	0.39	0.82	0.83	0.79	0.71
Gly	19.11	16.53	9.74	5.3	0.67	18.07
Ala	0.26	0.62	2.84	1.02	0.89	0.92
Tyr	1.69	0.83	1.42	0.9	0.89	1.27
Arg	0.88	0.92	1.12	0.77	0.9	1.01
Met	0.82	0.48	1.01	0.74	0.93	1.52
Val	0.52	0.81	0.95	1.33	0.84	0.92
Pro	0.9	0.8	1.82	0.83	0.82	0.88
Trp	0.54	0.89	0.59	0.52	0.57	0.67
Phe	0.86	0.74	2.66	1.66	1.07	1.66
Lys	0.57	0.21	2.07	1.65	0.26	0.68
Ile	0.91	0.74	0.92	0.82	0.79	2.75
Leu	0.82	2.61	2.45	0.72	0.73	4.98
